# A feature-efficient dual-task machine learning framework for predicting bone mineral density and osteoporosis stratification in resource-constrained environments

**DOI:** 10.1371/journal.pone.0354038

**Published:** 2026-07-20

**Authors:** Alina Maryum, Arslan Shaukat, Ehsan Yousaf, Ayesha Haque, Shazia Yusuf, Saif ul Haque, Humaira Ali, Soyiba Jawed, Muhammad Usman Akram

**Affiliations:** 1 Department of Computer and Software Engineering, National University of Sciences and Technology (NUST), Islamabad, Pakistan; 2 Department of Engineering, National University of Sciences and Technology (NUST), Islamabad, Pakistan; 3 Department of Anatomy, National University of Sciences and Technology (NUST), Islamabad, Pakistan; 4 Capital Hospital (CDA), Islamabad, Pakistan; 5 Department of Nuclear Medicine (AECH-NORI), Islamabad, Pakistan; 6 Department of Anatomy, Swat Medical College, Swat, Pakistan; University of Vermont, UNITED STATES OF AMERICA

## Abstract

Osteoporosis is a chronic skeletal disorder characterized by progressive bone mineral density (BMD) loss and structural deterioration, significantly increasing fracture risk. Despite its high prevalence, early detection remains challenging due to its asymptomatic progression and the limitations of conventional diagnostic techniques, such as Dual-Energy X-ray Absorptiometry (DXA). While DXA remains the clinical benchmark for BMD assessment, its high cost, limited accessibility, and inability to directly detect vertebral fractures necessitate the development of alternative, cost-effective, and widely deployable diagnostic methodologies. A dataset of 159 patient records was collected from NORI and CDA Hospital, incorporating 17 input features spanning demographics, genetic/blood type, clinical history and lab tests parameters. To bridge this gap, we developed a practical machine learning model tailored for clinics with limited resources. Instead of relying on expensive imaging, our framework uses only basic, highly accessible clinical markers—specifically ABO blood groups, serum calcium, and potassium levels. Because these tests are inexpensive and easily processed in standard laboratories, our approach removes the financial and technical hurdles of advanced diagnostics, making early screening possible in remote or underfunded healthcare settings. Data preprocessing involved rigorous feature selection, standardization, hyper-parameters tuning, clinically relevant features derivation and biomarker combinations. For classification, ensemble voting classifier was trained on key biomarkers— Weight, Potassium, Calcium and Total Vitamin D—achieving an accuracy of 90% and an AU-ROC score of 0.93 in predicting osteoporosis severity. In parallel, extreme gradient boosting Regressor trained on Age, Weight, ABO Group and Total Vitamin D demonstrated an R^2^ of 0.536 for lumbar spine BMD estimation. The proposed framework demonstrates the viability of leveraging machine learning for non-invasive osteoporosis screening and fracture risk assessment, offering a radiation-free and clinically accessible complementary pre-screening tool.

## Introduction

Osteoporosis is a critical global public health challenge, affecting millions of individuals worldwide, particularly older adults. This systemic skeletal disorder is characterized by a progressive reduction in bone mineral density (BMD) and the deterioration of the microarchitecture of bone tissue, ultimately leading to increased bone fragility and a higher risk of fractures [1]. Among the various osteoporotic fractures, vertebral fractures (VFs) are the most prevalent. However, their clinical detection is often challenging as they frequently occur without noticeable symptoms, earning them the moniker of “silent” fractures [2]. To detect osteoporosis, the gold-standard diagnostic tool, dual-energy X-ray absorptiometry (DXA), is widely used. However, DXA has several limitations, including high costs, limited accessibility, and an inability to directly detect vertebral fractures [3].

With recent advancements in artificial intelligence (AI) and deep learning (DL), medical imaging analysis has undergone a significant transformation, offering promising solutions for improving the detection and management of osteoporosis. In particular, convolutional neural networks (CNNs) have demonstrated remarkable efficiency in identifying abnormalities in medical images [4]. Although much research has been done in the field of osteoporosis detection using AI driven approach, the application of deep learning models to spine radiographs for the direct detection of both osteoporosis and vertebral fractures remains unexplored [5]. Additionally, many existing studies are hindered by small datasets, limited external validation, and an emphasis on BMD estimation rather than direct detection of vertebral fractures, which independently contribute to overall fracture risk [6]. Table 1 shows that serveral studies have explored CNNs for feature extraction and classification. However, challenges such as high computational costs, the need for large annotated datasets, and model interpretability remain areas for improvement.

**Table 1:** Comparative Analysis of Deep Learning Models for Osteoporosis Detection.

Study	Model Used	Preprocessing	Dataset	Results	Classification Type
Sukegawa et al. (2022) [7]	ResNet-152, EfficientNet-B7	Manual Cropping, Augmentation	778 Radiographic Images	AUC: 92.1%, F1-score: 74%	Binary
Wani et al. (2023) [8]	AlexNet, VGG-16, VGG-19, ResNet-18	Augmentation	381 Knee X-ray images	Accuracy: 91%	Multiclass
Mohammed et al. (2023) [9]	ResNet, U-GCN	Localization & Classification	1217 CT Images	AUC: 94.5%, Accuracy: 79.56%	Binary
Yamamoto et al. (2024) [10]	CNN, SVM, Random Forests	Data Extraction	11,369 Participants Chest X-ray and CT scans	AUC: 96%, Accuracy: 91%	Binary
Chen et al. (2023) [11]	ResNet50, Two-Level SVM	Radiomic Texture Analysis	197 LDCT Patients	Accuracy: 72%	Multiclass

Imaging-based models can capture structural patterns well, but these features often need to be complemented by additional information such as clinical features to improve diagnostic reliability as such features significantly influence bone health. Osteoporosis related bone loss is affected majorly with increasing age [2], low BMI [12], gender [13], lifestyle factors such as smoking [14], and sedentary lifestyle habits [15]. Other modifiable factors, including excessive alcohol consumption, poor diet, and insufficient intake of calcium and vitamin D, further exacerbate osteoporosis risk. Deficiencies in these nutrients can trigger secondary hyperparathyroidism, increase bone resorption, and lead to lower BMD levels. Furthermore, individuals with a history of fractures are at an elevated risk of future fractures which underscores the importance of early intervention in high-risk populations [16]. Another critical dimension in osteoporosis assessment involves clinical blood test parameters, such as complete blood count (CBC) data, which offer additional indicators of bone health. Several hematological markers have been linked to osteoporosis, including low hemoglobin levels, which may signify frailty and overall poor health, thereby increasing susceptibility to fractures [6]. Furthermore, elevated inflammatory markers have been associated with increased bone loss, as chronic inflammation plays a pivotal role in osteoclast-mediated bone resorption [17].

Studies utilizing ML models for osteoporosis detection are summarized in Table 2. By leveraging large datasets and advanced machine learning algorithms, predictive models can be developed that combine imaging-derived features with clinical risk factors to offer a more comprehensive evaluation of bone health [16]. Though ML models are effective in osteoporosis classification, they require extensive feature selection and engineering, which can introduce biases and limit model generalizability.

**Table 2:** Machine Learning Approaches for Osteoporosis Detection with Algorithms, Features, and Performance Metrics.

Study	ML Model Used	Preprocessing	Dataset	Results	Classification Type
V.V. Khanna et al. (2023) [18]	RF, Feature Selection	Feature median, SMOTE	1537 Observations	AUC: 0.94, F1-score: 89%, Accuracy: 89%	Binary
Hung et al. (2024) [19]	ANN, Linear Regression	Clinical Data Normalization	10,876 Women (DXA-Scans)	AUC: 0.913, Accuracy: 83.5%	Binary
R. Sebro et al. (2022) [20]	SVM	Clinical factors & Volumetric segmentation of each bone	163 patients	AUC: 0.702	Binary
K. Miura et al. (2024) [21]	Ridge, SVM, Random Forest, Gradient Tree Boosting	Clinical factors	201 patients	AUC: 0.853, r = 0.512%	Binary
P. Wändell et al. (2025) [22]	Stochastic Gradient Boosting	Clinical factors	30,741 patients	AUC: 0.95 (Women greater than 65 yrs.)	Binary

### Datasets utilized in osteoporosis research

Several public and private datasets are available for osteoporosis detection and classification. These datasets include medical images, clinical observations, and annotated records presented in Table 3.

**Table 3:** Publicly Available Datasets for Osteoporosis Research.

Dataset	Source	Data Type	Sample Size
**Mendeley Database**	Public	Hip radiographs, Knee X-rays and Spine DEXA Scans	Hip:128, Knee:240, Spine:177
**Kaggle**	Public	Knee X-ray Images	744 Images
**Harvard Dataverse**	Public	Osteoporosis Observations	1,573 Observations with 40 Features
**Hong et al. (2023)**	Private	Lateral Spine X-rays	26,299 X-rays from 9,276 patients
**Hung et al. (2024)**	Private	Clinical Data	10,876 Women

### Comparative analysis of diagnostic methods

Despite significant progress in AI-based osteoporosis detection, several challenges remain:

- **Multimodal Data Integration:** Combining imaging data with clinical and genetic information can enhance diagnostic accuracy.
- **Explainability and Interpretability:** Developing explainable AI models to increase trust in automated systems.
- **Severity Classification:** Most existing studies focus on binary classification; finer granularity in severity assessment is needed.
- **Real-Time Deployment:** Optimizing models for real-world clinical integration with low-latency inference.

Existing frameworks often rely on high-dimensional clinical data that are not available in primary care or resource-constrained settings. Furthermore, most studies focus exclusively on either classification or regression, missing the opportunity for a multi-stage parallel diagnostic approach. Our study fills this critical gap by proposing a lightweight, cost-effective and dual-stage machine learning framework validated on a self-collected dataset of 159 patients to perform multi-class classification and BMD estimation.

## Materials and methods

### Dataset

**Fig 1:**
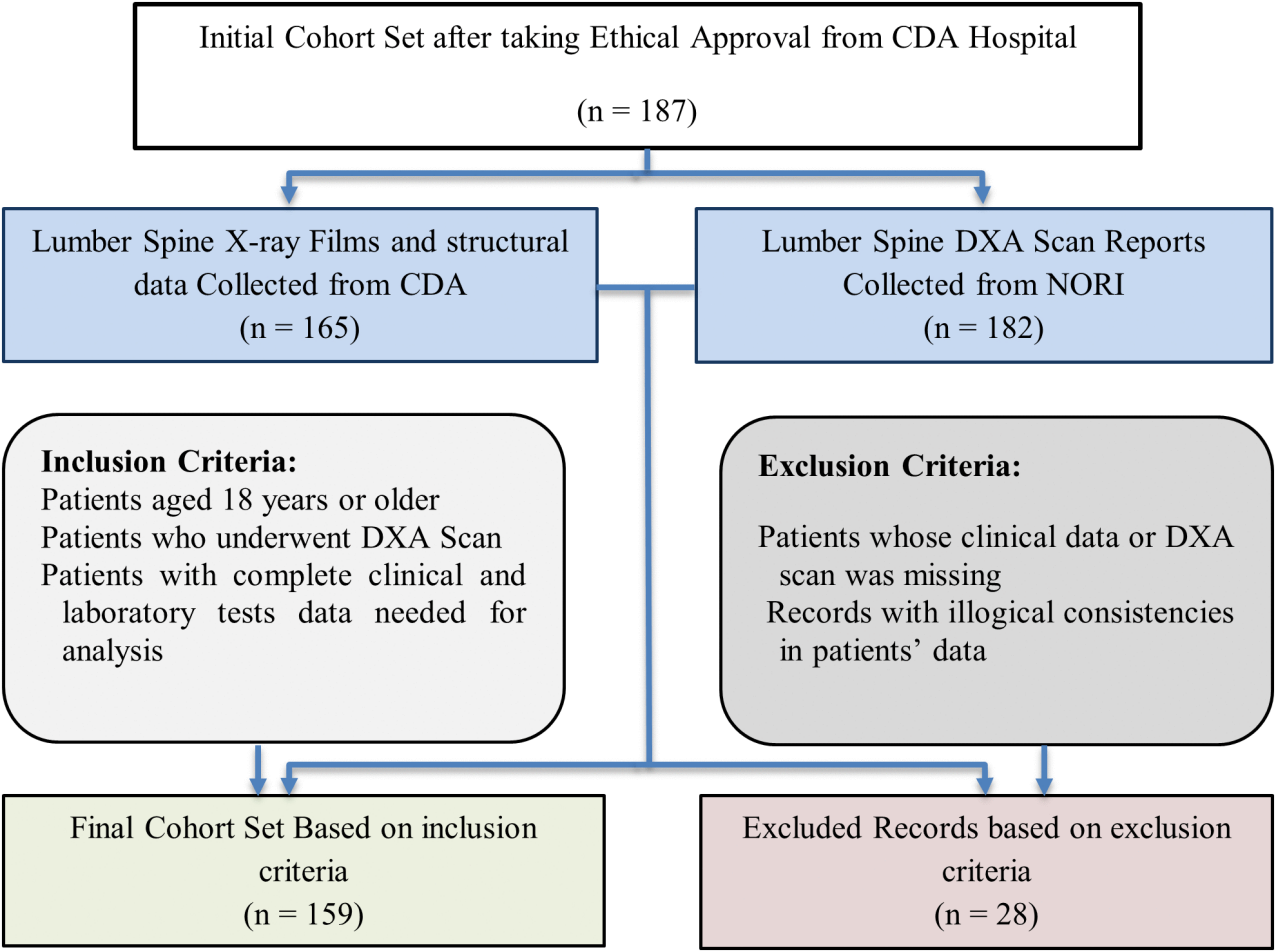
Data Acquisition Protocol. Detailed Workflow of Data Acquisition Protocol.

Data for this study were retrieved from the electronic health records (EHRs) of Nuclear Medicine Oncology and Radiotherapy Institute (NORI) and Capital Development Authority (CDA) hospitals, Pakistan. The recruitment period for dataset collection was from 01/07/2024–30/12/2025. Institutional Research Board and Ethical Committee, CDA Islamabad granted full ethical approval for the study protocol which covered the use of de-identified patient data. Informed written consent was obtained from all willing participants. The data were accessed from EHR for analysis after complete acquisition from 05/01/2026–25/02/2026. To maintain patient confidentiality and comply with ethical standards, all data was anonymized before the analysis. All direct patient identifiers, such as names, and medical record numbers, were removed. Each patient was assigned a unique, random identifier to maintain data linkage without compromising confidentiality. This study’s population included a cohort of 187 adults among them 77 were male and 110 were female. Participants were adults aged 18 years and older who had undergone dual energy X-ray absorptiometry (DXA) scan with a T-score recorded for the lumbar spine. Individuals with a prior diagnosis of metabolic bone disease other than osteoporosis were removed from the analysis. A comprehensive data collection protocol is illustrated in Fig 1. Only those records of the patients were included that were complete in all aspects (including DXA scan and all biomarkers data). The final dataset contain the records of 159 patients who underwent X-Ray of lumbar spine for various reasons excluding fractures and bone tumors or history of previous spine trauma. Based on the aim of the study, following 17 variables were included: (a) Demographic data- Age and Gender (b) Anthropometric measurements- Weight, Height and Body Mass Index (BMI) (c) History Information- Smoking, Trauma, Diabetes, Menopause (d) Laboratory tests- ABO-Blood group, Rh factor: positive or negative, Hemoglobin (Hb), Hematocrit (HCT), Alkaline Phosphatase, Serum Potassium, Serum Calcium and Vitamin D levels. Apart from the input biomarkers, we also collected the data of L1 to L4 Lumbar Spine Bone Mineral Density (BMD) value obtained through DXA. Based on the value of BMD, patients were categorized as normal, osteopenic or osteoporotic.

Among the 2 target variables, L1 to L4 Lumber Spine BMD has a continuous output value ranging from 0.476 to 1.545 g/cm3. The second response variable was used to determine the severity of osteoporosis. For that three discrete labels were used; 0 for Normal, 1 for osteopenia, and 2 for osteoporosis. The dataset collected was imbalanced being biased towards the normal case having 49.06% of the samples, osteopenia has 34.59% and osteoporosis, the minority class has 16.35% of the total samples.

### Proposed methodology

**Fig 2:**
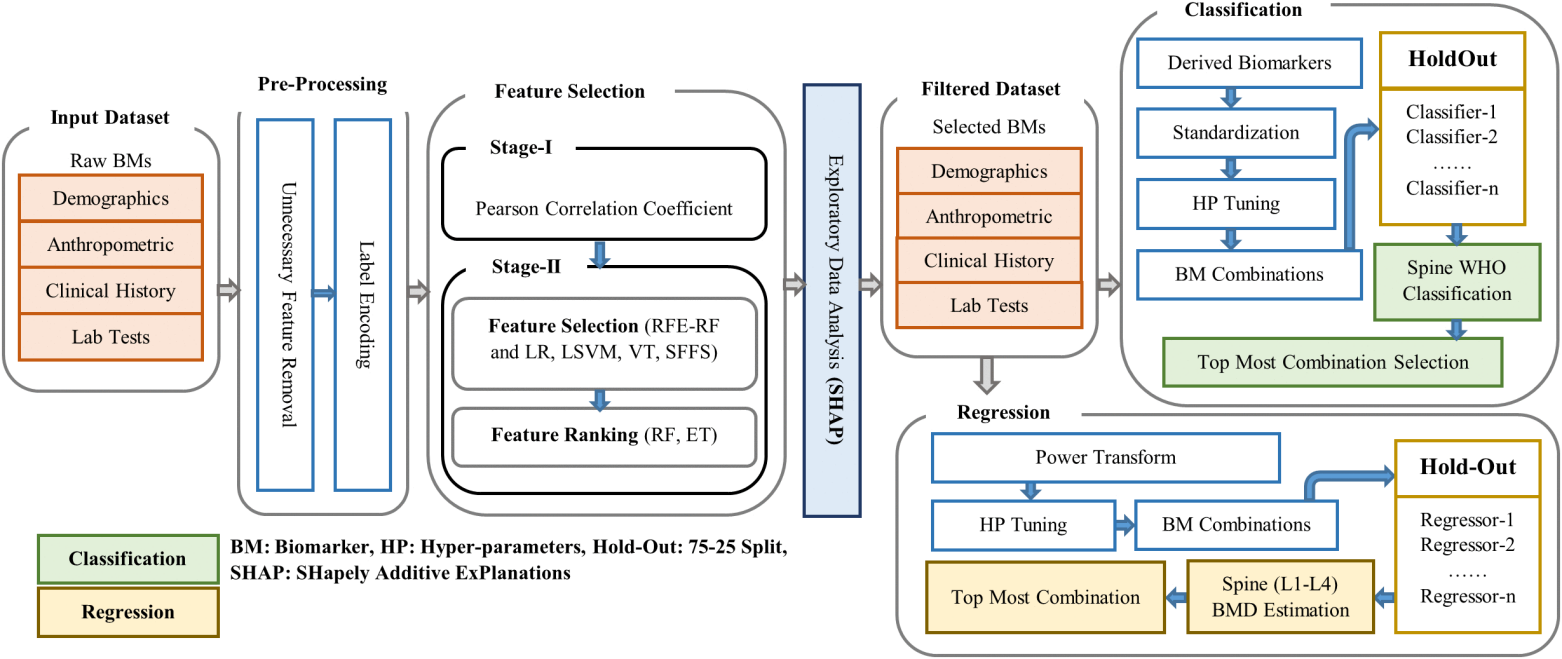
Proposed Methodology. Detailed Workflow of Proposed Methodology for WHO Spine Classification and BMD Estimation.

The numerical raw data were pre-processed and optimal features were filtered and ranked using various machine learning models. A collection of machine learning classifiers and regression models were selected and exhaustively trained separately for each of the input biomarkers combinations. The overall workflow of the proposed methodology is shown in Fig 2. Each of the step performed is explained in detail in below sub-sections.

#### Data pre-processing.

The raw data collected from the hospital were first pre-processed using various steps. First, unnecessary features that don’t contain any useful information for predicting the output variable were identified and removed. Few values for certain biomarkers that were missed during data collection, were filled using class mean. After that, label encoding was applied on the categorical features for transforming the features to their equivalent numerical values for further processing.

#### Feature selection.

The pre-processed data were used as input for feature selection to select the most relevant and optimal features. This process was carried out in two stages.

In Stage-I, Pearson Correlation Coefficient Matrix was used to analyze the relationship between input variables and to check for multicolinearity among them using Eq (1).


$$\begin{array}{c} r=\frac{\sum (x_{i}-\bar{x})(y_{i}-\bar{y})}{\sqrt{\sum (x_{i}-\bar{x}{)}^{2}\sum (y_{i}-\bar{y}{)}^{2}}} \end{array}$$ (1)

where *r* is the correlation coefficient value within the range −1–1. $x_{i}$ and *x* are a single instance value and mean value of the first input variable, respectively, and $y_{i}$ and *y* are a single instance value and mean value of the second input variable, respectively. We choose a threshold value of 0.75, as a result the most highly correlated features were removed. The same Correlation Matrix was also visualized using Correlation Heatmap by utilizing python seaborn library.

In Stage-2, the selected features from Stage-1 were further filtered using 5 feature selection techniques including Sequential Forward Floating Selection (SFFS), Linear Support Vector Machine (LSVM), Variance Threshold (with a threshold value of 0.1), Recursive Feature Elimination using Random Forest (RFE-RF) and Logistic Regression (RFE-LR). After that, features were ranked using 2 feature ranking techniques including Extra Trees (ET) and Random Forest (RF) based on their importance. At the end, 10 top most features were selected for further analysis.

#### Exploratory data analysis.

**Fig 3:**
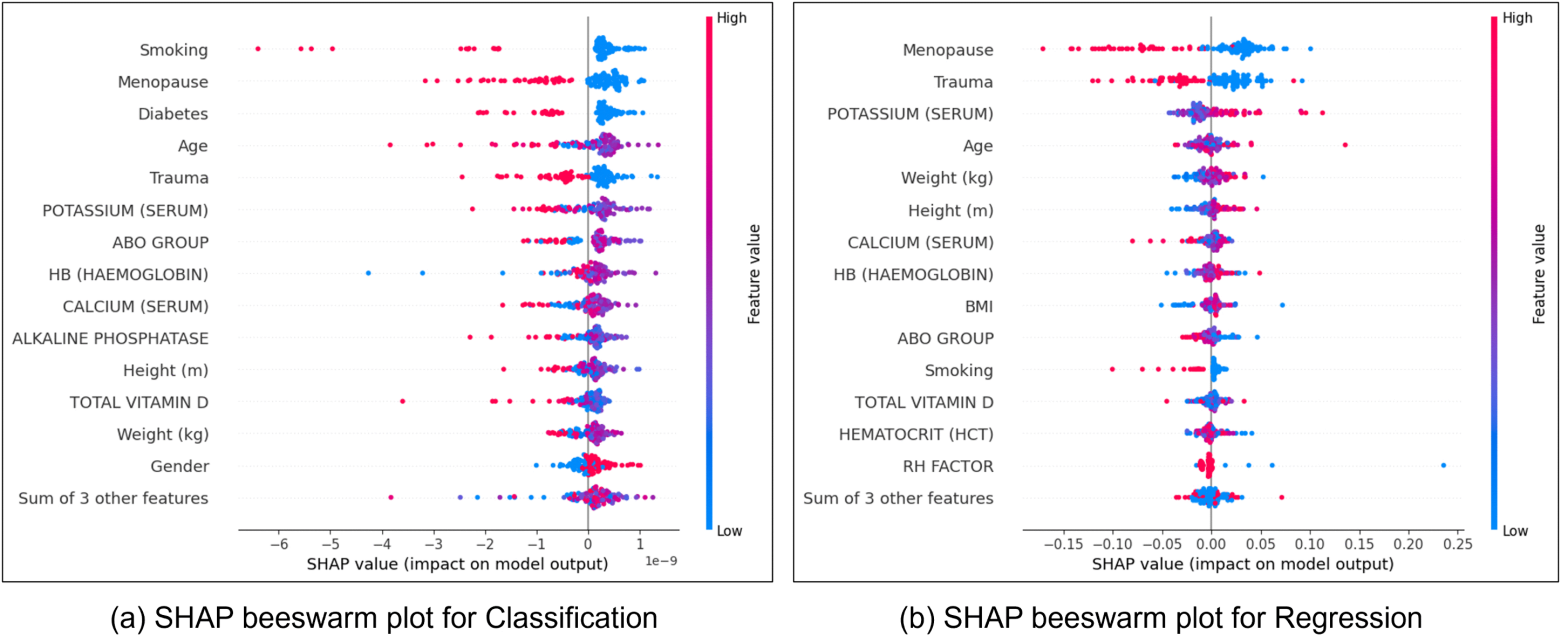
SHapely Additive ExPlanations (SHAP) Plots. SHAP Analysis with beeswarm plot using Extra Trees showing the impact of each biomarker for the final Osteoporosis Severity Level Prediction (Left) and BMD Estimation (Right) in descending order of their importance.

For further feature filtering to obtain the optimal features, SHapely Additive ExPlanations (SHAP) analysis was used together with domain knowledge. Fig 3 shows feature importance based on their SHAP values using Extra Trees Classifier and Regressor, and how each feature contributes towards the severity level prediction of osteoporosis and BMD estimation, respectively. Based on the analysis, 8 features were selected as a final feature set ($X_{c}$) for classification and 9 features were selected as a final feature set ($X_{r}$) for regression.

#### Data split.

For classification, data with the final feature set (*X*_class_) were divided into training and test subsets using Hold-out technique in the ratio of 75:25 for training and testing purpose.

#### Standardization and power transform.

The final feature set obtained was standardized using the StandardScalar provided by python scikit-learn library for the classification task. As the input features had different scales, this preprocessing technique removed bias by scaling all the features to 1 and removing the mean, given by Eq (2).


$$\begin{array}{c} z_{i}=\frac{x_{i}-\mu_{i}}{\sigma_{i}} \end{array}$$ (2)

Where $z_{i}$ is the normalized input feature vector, $x_{i}$ is the pre-normalized input feature vector, and $\mu_{i}$ and $\sigma_{i}$ are the mean and standard deviation of the $i^{th}$ feature vector values, respectively.

For regression, along with standardization, Power Transform was also applied using the Yeo-Johnson method to transform the shape of data just like a Gaussian distribution. The response variable was also transformed. It improves the overall performance of the model by reducing skewness and variance in the data.

Yeo-Johnson transform for the $i^{th}$ input feature $x_{i}$ is given by Eq (3).


$$\begin{array}{c} Z(x_{i})=\left\{ \begin{array}{cc} \frac{(x_{i}+1{)}^{\lambda }-1}{\lambda }, & \text{if }\lambda \neq 0,x_{i}\geq 0\\ \log(x_{i}+1), & \text{if }\lambda =0,x_{i}\geq 0\\ \frac{-((|x_{i}|+1{)}^{2-\lambda }-1)}{2-\lambda }, & \text{if }\lambda \neq 2,x_{i}<0\\ -\log(|x_{i}|+1), & \text{if }\lambda =2,x_{i}<0 \end{array}\right. \end{array}$$ (3)

where λ is an automatically learned parameter to stabilize the variance, and $Z(x_{i})$ is the transformed *i*th feature vector.

#### Data imbalance handling.

As discussed above, the data set was imbalanced, being biased towards normal class with a very small proportion of samples in the osteoporosis class. To handle class imbalance, we used class weights to penalize misclassifications from minority class. Though there are other options available as well to balance the dataset including SMOTE and its variants, ADASYN and GAN based methods, these are distribution-learning methods for oversampling. With a very small size of minority class (only 20 samples for osteoporosis), these methods fail to learn the distribution and generate noisy, duplicate and overlapping samples destroying the class boundaries. So, for our dataset, we used class weights to reduce bias towards the majority class and pay more attention to the minority class, thereby improving recall and hence the overall performance.

#### Hyper-parameters tuning.

To improve the performance of machine learning models, optimal hyper-parameters need to be selected. As the dataset size was limited, Grid Search was used to select the optimal hyper-parameters. A list of manually selected hyper-parameters were exhaustively checked against each classifier and regressor and the best ones were selected that were further used to train various machine learning models.

#### Classification.

Finding the severity level of osteoporosis was a multi-class classification problem as there were three discrete output labels. Given this, the selection of meaningful and discriminative features becomes crucial for improving classification performance. So, the most relevant biomarkers (Calcium, Potassium and Vitamin D levels) were used to derive three more features from them including Total Vitamin D to Calcium ratio, Potassium to Calcium ratio and Total vitamin D-Potassium interaction because they represent clinically meaningful relationships related to bone metabolism. Further, 10 different machine learning classifiers were trained using the optimal hyper-parameters already selected. Machine learning models were chosen from linear, non-linear, tree-based and ensemble learning categories and included Logistic Regression (LR), k-Nearest Neighbors (k-NN), Support Vector Machine (SVM), Random Forest (RF), Gradient Boosting (GB), Extreme Gradient Boosting (XGBoost), Categorical Boosting (CatBoost), Adaptive Boosting (AdaBoost), Extra Trees (ET), and Ensemble Voting that used soft voting among the top 3 classifiers. We checked all possible combinations of the selected biomarkers with feature set size ranging from 3 to 8. All the combinations of features were exhaustively trained separately against each of the above-mentioned classifier.

#### Regression.

Finding the Lumber Spine BMD value was a regression task as the output label was a continuous value. For regression, 10 different machine learning regression models were trained using the optimal hyper-parameters already selected for this task. Machine learning models were chosen from linear, non-linear, and tree-based learning categories and included RANdom SAmple Consensus (RANSAC), Ridge, Elastic Net, Least Absolute Shrinkage and Selection Operator (LASSO), k-Nearest Neighbors (k-NN), Support Vector Machine (SVM), Extra Trees (ET), Gradient Boosting (GB), Extreme Gradient Boosting (XGB) and Adaptive Boosting (AdaBoost). We checked all possible combinations of the selected biomarkers with feature set size ranging from 3 to 8. All the combinations of features were exhaustively trained separately against each of the above-mentioned regression model.

#### Performance metrics.

For classification, five Performance Evaluation Metrics (PEM) were used including Accuracy, F1-score and Area Under Receiver Operating Characteristics Curve (AU-ROC) score measured using Confusion Matrix that details the exact number of true positive (TP), true negative (TN), false positive (FP) and false negative (FN) samples in the form a matrix. For regression, we used three PEMs including Root Mean Square Error (RMSE), Mean Absolute Error (MAE), and R-Squared (*R*^2^) values. Their mathematical definitions, operational focus, and ideal target boundaries are consolidated in Table 4.

**Table 4:** Summary of Performance Evaluation Metrics.

Metric	Mathematical Formula	Measurement Focus	Ideal Value
Accuracy	$\frac{TP+TN}{TP+TN+FP+FN}$	Overall classification correctness	1.0
F1-Score	$2\times \frac{\text{Precision}\times \text{Recall}}{\text{Precision}+\text{Recall}}$	Harmonic mean of precision and recall	1.0
${\text{AU-ROC}}_{i}$	$\frac{1}{2}-\frac{FP}{2(FP+TN)}+\frac{TP}{2(TP+FN)}$	Individual class diagnostic trade-off	1.0
AU-ROC	$\frac{1}{N}\sum_{i=1}^{N}{\text{AU-ROC}}_{i}$	Macro-average area under the curve	1.0
RMSE	$\sqrt{\frac{1}{N}\sum_{i=1}^{N}(y_{i}-{\hat{y}}_{i}{)}^{2}}$	Variance of large prediction errors	0.0
MAE	$\frac{1}{N}\sum_{i=1}^{N}|y_{i}-{\hat{y}}_{i}|$	Average absolute error magnitude	0.0
*R* ^2^	$1-\frac{\sum_{i=1}^{N}(y_{i}-{\hat{y}}_{i}{)}^{2}}{\sum_{i=1}^{N}(y_{i}-{\bar{y}}_{i}{)}^{2}}$	Proportion of explained variance	1.0

TP: True Positive, TN: True Negative, FP: False Positive, FN: False Negative, $y_{i}$: Actual value, ${\hat{y}}_{i}$: Predicted value, ${\bar{y}}_{i}$: Mean of actual values, and *N*: Number of total samples.

## Results

The results obtained, based on the exhaustive training and validation, are presented in the form of biomarkers combinations. For classification, the best combination is defined as a subset of biomarkers within the above defined range against which accuracy as well as f1 score is the highest. Similarly, for regression, the best combination is defined as a subset of biomarkers against which *R*^2^ is the highest. Table 5 contains the details of each of the biomarker that was collected and analyzed in this study in terms of their category, type, mean and standard deviation values and the class distribution.

**Table 5:** Details of the Osteoporosis-Biomarkers Dataset Collected, Used and Analyzed in this Study.

Category	Biomarkers (unit)	Type	Mean ± Std.	Distribution: n(%)
Demographics	Age (years)	Numerical	50.67 ± 13.67	–
	Gender	Categorical	–	Male: 62 (38.99%)
				Female: 97 (61.01%)
Anthropometric	Height (m)	Numerical	1.62 ± 0.1	–
	Weight (kg)	Numerical	71.16 ± 14.12	–
	BMI (kg/m^2^)	Numerical	27.1 ± 5.09	–
Clinical History	Smoking	Categorical	–	Yes: 21 (13.46%)
				No: 135 (86.54%)
	Menopause	Categorical	–	Pre-M: 100 (64.52%)
				Post-M: 55 (35.48%)
	Trauma	Categorical	–	Yes: 66 (42.31%)
				No: 90 (57.69%)
	Diabetes	Categorical	–	Yes: 41 (26.8%)
				No: 112 (73.2%)
Lab tests	Hb (g/L)	Numerical	13.03 ± 1.85	–
	HCT (%)	Numerical	40.22 ± 4.54	–
	ABO Group	Categorical	–	A: 26 (16.46%)
				B: 59 (37.34%)
				AB: 49 (31.01%)
				O: 24 (15.19%)
	Rh Factor	Categorical	–	Rh-N: 6 (4.05%)
				Rh-P: 142 (95.95%)
	Serum Potassium (mmol/L)	Numerical	4.24 ± 0.38	–
	Alkaline Phosphatase (U/L)	Numerical	233.54 ± 72.39	–
	Vitamin D Level (nmol/L)	Numerical	44.52 ± 32.02	–
	Serum Calcium (mmol/L)	Numerical	9.23 ± 0.79	–

Hb: Hemoglobin, HCT: Hematocrit, Rh-N: Rhesus antigen Negative,Rh-P: Rhesus antigen Positive,Pre-M: Pre-Menopause, Post-M: Post-Menopause.

Table 6 shows the top combinations against each of subset ranging from three to eight biomarkers. The corresponding experimentation results of classification are presented in Table 7. Table 8 shows the confusion matrix of the top most combination in terms of highest accuracy and Table 9 presents the results against each regression biomarker combination in terms of RMSE, MAE and *R*^2^ value.

**Table 6:** Top Biomarkers combinations in each of the subset range for classification and regression response variables.

Model	Biomarker’s Combinations	NoF	Comb. Code
**a) Spine WHO Classification**			
EV	Calcium, Vitamin D/Calcium, Vitamin D-Potassium	3	SC1
EV	Calcium, Potassium/Calcium, Total Vitamin D, Weight	4	SC2
EV	Calcium, Potassium/Calcium, Vitamin D/Calcium, Weight, Potassium	5	SC3
EV	Calcium, Total Vitamin D/Calcium, Alkaline Phosphatase, Weight, Age, Trauma	6	SC4
EV	Calcium, Potassium/Calcium, Vitamin D/Calcium, Alkaline Phosphatase, Trauma, Total Vitamin D, Diabetes	7	SC5
EV	Calcium, Potassium/Calcium, Vitamin D-Potassium, Potassium, Total Vitamin D, Diabetes, Smoking, Weight	8	SC6
**b) Lumber Spine (L1-L4) BMD Estimation**			
XGB	Age, Weight, Total Vitamin D	3	BE1
XGB	Age, Weight, Total Vitamin D, ABO Group	4	BE2
XGB	Age, Weight, Total Vitamin D, ABO Group, Diabetes	5	BE3
XGB	Age, Weight, Total Vitamin D, ABO Group, Diabetes, Smoking	6	BE4
XGB	Age, Weight, Total Vitamin D, ABO Group, Diabetes, Smoking, Alkaline Phosphatase	7	BE5
XGB	Age, Weight, Total Vitamin D, ABO Group, Diabetes, Smoking, Alkaline Phosphatase, Menopause	8	BE6

SVM: Support Vector Machine, EV: Ensemble Voting, GB: Gradient Boosting, NoF: No. of Features, Comb: Combinations Code, SC: Spine Classification, BE: BMD Estimation

**Table 7:** Spine WHO Classification Results in terms of Accuracy, F1-score and aggregated AU-ROC value based on the top selected combination against each subset range.

Combination Code	Accuracy	F1-Score	AU-ROC
SC1	0.8	0.8	0.89
SC2	0.9	0.9	0.93
SC3	0.85	0.85	0.92
SC4	0.85	0.85	0.84
SC5	0.88	0.88	0.87
SC6	0.90	0.90	0.91

**Table 8:** Confusion Matrix showing Spine Classification against best four biomarkers combination of Weight, Potassium, Total Vitamin D and Calcium.

	Predicted Class		
Actual Class	Normal	Osteopenia	Osteoporosis
**Normal**	19	1	0
**Osteopenia**	1	13	0
**Osteoporosis**	0	2	4

**Table 9:** Lumber Spine (L1-L4) BMD Estimation Results in terms of RMSE, MAE, and R^2^ Score based on the top selected combination against each subset range.

Combination Code	RMSE	MAE	R^2^ Value
BE1	0.121	0.096	0.418
BE2	0.108	0.085	0.536
BE3	0.111	0.086	0.515
BE4	0.113	0.087	0.5
BE5	0.122	0.098	0.405
BE6	0.131	0.106	0.321

## Discussion

### Spine WHO classification

#### Optimal feature subset.

The top 9 features included one Demographic (Age), one anthropometric (Weight (kg)), three clinical (Smoking, Trauma, Diabetes) and three lab tests (Vitamin D level, Serum Potassium and Serum Calcium).

#### Proposed combination.

The results presented in Table 7 shows that SC2, SC6, and SC5 produced the best results giving 90%, 90% and 88% accuracy score and 0.93, 0.91 and 0.87 AUC-ROC score, respectively using ensemble voting classifier. Among these three biomarkers Combination, SC2 gave the highest accuracy score containing Weight, Serum Potassium, Total Vitamin D level and Serum Calcium. Results show that these four chosen biomarkers contribute the most towards predicting the severity level of osteoporosis using minimal resources and cost giving an overal accuracy of 90%. Confusion matrix results as given in Table 8 against the best combination show that the ensemble voting classifier was able to correctly classify 95% normal, 93% osteopenia, and 67% of osteoporotic cases.

**Fig 4:**
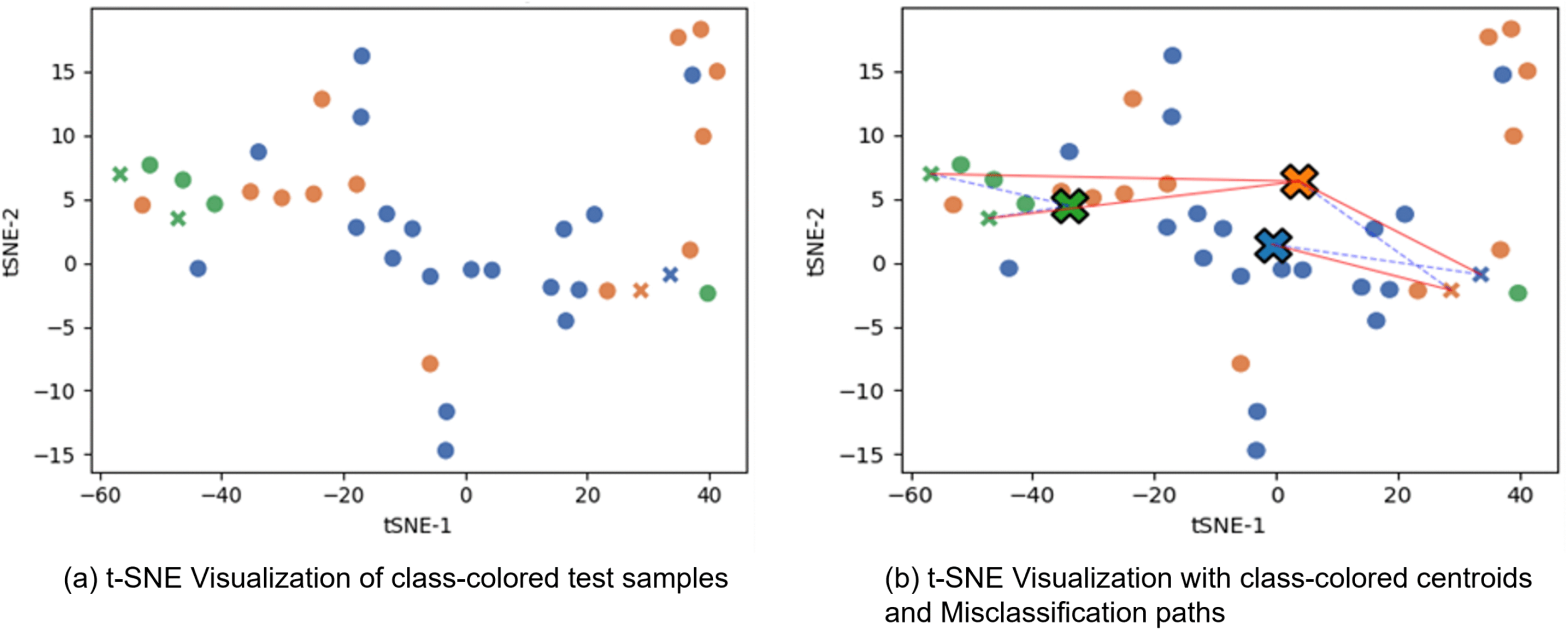
t-SNE Visualization of Test Samples. t-SNE Visualization of class colored test samples (Blue: Normal, Orange: Osteopenia, Green: Osteoporosis) with misclassified samples highlighted by a cross symbol (Left) and class colored centroids represented by bold colored crosses and Misclassification paths (Right).

As clear from the confusion matrix, the model misclassified 1 sample of normal class as osteopenia, 1 sample of osteopenia as normal and 2 samples of osteoporosis as osteopenia. For these 4 misclassified samples, we checked the similarity of the selected input features with the predicted classes, shown in Table 10a using class centroids, shown in Table 10b. Based on Euclidean Distance, we analyzed the distance of misclassified samples with the actual class as well as the predicted class. Table 10c shows that the the distance of misclassified samples to the centroid of predicted class is less as compared to the centroid of actual class. This is because the misclassified samples input feature values resembled those of the predicted class input features. That’s why the model incorrectly labelled them as predicted class. The t-SNE plot of the test samples along with misclassified samples is shown in Fig 4a and class centroids along with the misclassification path is shown in Fig 4b.

**Table 10:** Comprehensive Analysis of Misclassified Samples and Class Centroids.

(a) Misclassified Samples input feature values, actual class label and predicted class label in Test Dataset					
**Calcium**	**Potassium/Calcium**	**Total Vitamin D**	**Weight (kg)**	**Actual Class**	**Predicted Class**
8.9	0.438	20.0	42	Osteoporosis	Osteopenia
9.4	0.436	23.0	55	Osteoporosis	Osteopenia
9.2	0.435	67.0	95	Osteopenia	Normal
9.1	0.451	77.0	77	Normal	Osteopenia
(b) Class Centroids of three output classes against each selected input feature in Train Dataset					
**Class Label**	**Calcium**	**Potassium/Calcium**		**Total Vitamin D**	**Weight (kg)**
Normal	9.184	0.471		45.571	74.103
Osteopenia	9.344	0.446		46.561	69.731
Osteoporosis	9.185	0.465		51.792	66.7
(c) Misclassification Distance Analysis Table showing the distance of misclassified samples with the centroids of actual and predicted classes					
**Actual**	**Predicted**	**Distance to Actual**		**Distance to Predicted**	**Closer to**
**Class**	**Class**	**Class Centroid**		**Class Centroid**	**Class?**
Osteoporosis	Osteopenia	40.26		38.4	Osteopenia
Osteoporosis	Osteopenia	31.08		27.79	Osteopenia
Osteopenia	Normal	32.5		29.93	Normal
Normal	Osteopenia	31.56		31.3	Osteopenia

### Lumber Spine (L1-L4) BMD Estimation

#### Optimal feature subset.

The top 9 features included one demographic (Age), one anthropometric (Weight (kg)), three clinical (Smoking, Menopause, Diabetes), and four lab tests ((ABO Group, Vitamin D Level, Alkaline Phosphatase and Serum Calcium).

#### Proposed combination.

Ta The results presented in Table 9 shows that BE2, BE3 and BE4 produced the best results giving R^2^ value of 0.536, 0.515 and 0.5, respectively using Extreme Gradient Boosting. Among these combinations, BE2 is selected as the best one for regression task containing four input features; Age, Weight, ABO Group and Total Vitamin D giving R^2^ value of 0.536, RMSE of 0.108 and MAE of 0.085 for Spine BMD Estimation using Extreme Gradient Boosting. The results indicate that using these four biomarkers, the model was able to explain about 53.6% of the variance in the data. This moderate R^2^ value must be evaluated against its role as a preliminary triage tool. In remote or low-resource settings lacking advanced diagnostic imaging, a model that captures over 50% of BMD variance using routine laboratory markers provides a vital clinical baseline. Rather than acting as a definitive diagnosis, this level of accuracy serves to isolate high-risk individuals for early intervention, proving that effective risk stratification is possible even without expensive infrastructure. This moderate predictive strength is entirely comparable to established clinical risk frameworks like FRAX when used without BMD data. In [23]  the authors have demonstrated that optimal FRAX thresholds for predicting future fractures routinely operate at low numerical cut-offs (e.g., probability thresholds as low as 1.1% to 6.3% depending on sex) yet remain globally accepted and highly effective for population triage and early clinical intervention.

### Rationale for predictor selection

To maintain clinical utility in resource-limited settings, the framework pairs serum calcium and Total Vitamin D, the core elements of bone structure with secondary, easily obtainable laboratory markers. Potassium serves a protective, metabolic role rather than building bone directly. Adequate potassium levels help the body preserve its existing mineral matrix by reducing calcium loss and preventing the body from pulling calcium out of the skeleton [24]. Conversely, the ABO blood group is treated as an exploratory marker. Existing literature on the relationship between blood type and bone density is heavily divided. While some regional studies report a distinct correlation where specific blood groups exhibit higher rates of bone loss [25–27], other clinical trials found no statistical connection whatsoever [28,29]. Given these conflicting global findings, our framework avoids assuming any direct biological causation. Instead, blood type is used strictly as a zero-cost demographic indicator to help map localized risk trends where advanced diagnostic bone scans are unavailable.

### Performance evaluation of proposed framework

**Fig 5:**
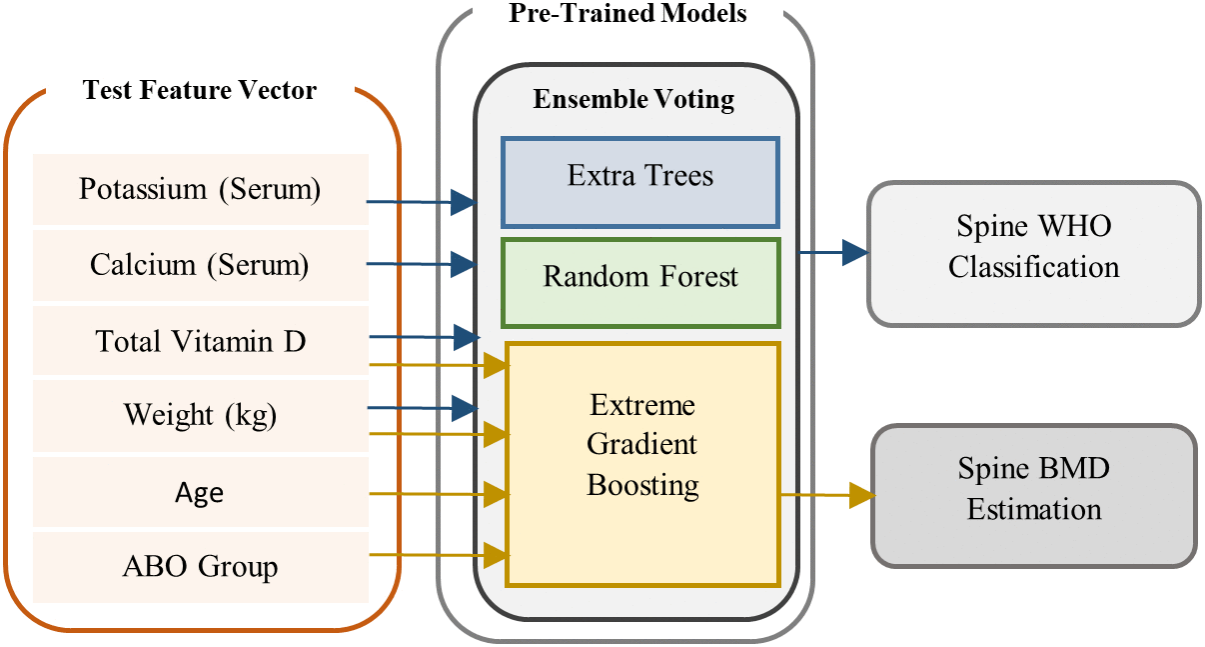
Proposed Protocol for Evaluation. Proposed Framework for Spine Classification using only a few biomarkers, providing an easily accessible and radiation free pre-screening tool.

Based on the above results and discussion, the proposed framework for Spine Classification and BMD Estimation is shown in Fig 5. Whenever a patient with back pain in lumber spine arrives at the hospital, four to six features need to be collected including Age (yrs), Weight (kg), ABO Group, Potassium (serum), Calcium serum (mmol/L) and Total vitamin D level depending on the required output (BMD prediction or Spine Classification). This set of biomarkers will be considered as the input features where Weight, Serum Potassium, Total Vitamin D level and Serum Calcium may be fed to Ensemble Voting Classifier. For regression, Age, Weight, ABO Group and Total Vitamin D level may be fed to Extreme Gradient Boosting Regressor for BMD estimation. This study offers a very economical, radiation-free and easily available way to detect osteoporosis without the need to undergo DXA, CT scan and MRI that require high radiation dose, are very costly and mostly inaccessible to the majority in rural or underdeveloped areas.

### Bench marking against previous studies

Table 11 shows the results of classification as well as regression with existing technique used in literature. Based on the results and various factors, it can be concluded that our proposed work produced the best multi-class classification results and satisfactory regression results even with small amount of input data (159 samples only) giving accuracy of 90% and AU-ROC score of 0.93 leading all the existing state-of-the-art work where tabular data is used. By applying various pre-processing on the data including feature selection and ranking, employing various exploratory data analysis, clinically derived features, standardization and exhaustive training of various feature combinations, can help improve the overall performance of ML models leading to efficient and accurate results.

**Table 11:** Comparison of Proposed Work with existing state-of-the-art techniques in terms of model used, pre-processing, dataset size and classification and Regression results.

Study	Model Used	Pre-processing	Dataset Size	Input Modality	Cl. Type	Acc (%)	F1	AUC	RMSE	R^2^
**Wani et al. [8]**	CNN, SVM, RF	Augmentation	381	Knee X-ray	Multi	91	–	–	–	–
**Yamamoto et al. [10]**	CNN, SVM, RF	Data Extraction	11,369	Chest X-ray and CT scans	Binary	91	–	0.96	–	–
**Khanna et al. [30]**	LR, DT, NB, SVM	SMOTE	1,493	Clinical Data	Binary	89	0.89	0.94	–	–
**Yasaka et al.** [4]	CNN	–	1,665	CT scans	Binary	–	–	0.96	–	0.85
**Hung et al. [19]**	ANN, LR	Normalization	10,876	Clinical Data	Binary	83.5	–	0.91	0.09	–
**Proposed Work**	Ensemble Voting	FS, SMOTE, HP tuning, Standardization	159	Clinical Data	Multi	90	0.9	0.93	0.1	0.53

Cl. Type: Classification Type, Acc. Accuracy, F1: F1-Score, AUC-AU-ROC Score, Multi: Multi-Class Classification, NB: Naïve Bayes, FS: Feature Selection, HP: Hyper-Parameters

### Study limitations

There are certain limitations to this study. First, the data used in this work is imbalanced and very limited in terms of the number of samples. For that, more data of such patients can be collected. Second, it is a single-center study, i.e., the data is collected from a single hospital within 12–14 months timespan. Therefore, it doesn’t capture the diversity and is only limited to a single demographic region with no external validation. Therefore, the model may not be able to correctly classify the patients’ data from other regions. Expanding this research to include a more diverse group of patients from multi-center settings would strengthen the reliability of the findings. Additionally, incorporating more clinical variables could refine the accuracy of the models and improve their predictive performance.

## Conclusion and future work

Osteoporosis continues to be a serious global health issue, affecting millions of people and increasing their risk of fractures, particularly as they age. While methods like Dual-Energy X-ray Absorptiometry (DXA) remain the standard for diagnosing low bone mineral density (BMD), these tests are expensive, not always accessible, and expose patients to radiation. This study explores a practical and cost-effective preliminary, exploratory screening tool by using a small set of clinical biomarkers combined with machine learning (ML) models to detect osteoporosis and estimate BMD. Building on the findings of our work, this study proposes a cost-effective and easily accessible diagnostic framework that could be easily implemented in hospitals and clinics. When a patient presents with symptoms like lower back pain, doctors could collect a small set of biomarkers such as Age, Weight, ABO Group, Calcium, Potassium and Total Vitamin D—and input them into trained ML models. These models would then predict osteoporosis severity and estimate BMD without requiring expensive imaging, making early detection more accessible to a broader population. To further validate and refine this approach, future studies should focus on collecting data from larger and more diverse populations across multiple hospitals. Additionally, integrating other clinical factors—such as genetic markers, lifestyle habits, and dietary patterns—could improve the models’ ability to predict osteoporosis risk more accurately. Further exploration of more advanced computational techniques, including deep learning models may also enhance diagnostic precision. Moreover, ensuring that these models are interpretable for healthcare professionals is essential for their real-world adoption. Finally, implementing this diagnostic framework in clinical settings could streamline osteoporosis screening and assist doctors in making more informed decisions.
